# Decon2LS: An open-source software package for automated processing and visualization of high resolution mass spectrometry data

**DOI:** 10.1186/1471-2105-10-87

**Published:** 2009-03-17

**Authors:** Navdeep Jaitly, Anoop Mayampurath, Kyle Littlefield, Joshua N Adkins, Gordon A Anderson, Richard D Smith

**Affiliations:** 1 Environmental Molecular Science Laboratory, Pacific Northwest National Laboratory, Richland, WA, 99352, USA; 2 Fundamental and Computational Sciences Directorate, Pacific Northwest National Laboratory, Richland, WA, 99352, USA

## Abstract

**Background:**

Data generated from liquid chromatography coupled to high-resolution mass spectrometry (LC-MS)-based studies of a biological sample can contain large amounts of biologically significant information in the form of proteins, peptides, and metabolites. Interpreting this data involves inferring the masses and abundances of biomolecules injected into the instrument. Because of the inherent complexity of mass spectral patterns produced by these biomolecules, the analysis is significantly enhanced by using visualization capabilities to inspect and confirm results. In this paper we describe Decon2LS, an open-source software package for automated processing and visualization of high-resolution MS data. Drawing extensively on algorithms developed over the last ten years for ICR2LS, Decon2LS packages the algorithms as a rich set of modular, reusable processing classes for performing diverse functions such as reading raw data, routine peak finding, theoretical isotope distribution modelling, and deisotoping. Because the source code is openly available, these functionalities can now be used to build derivative applications in relatively fast manner. In addition, Decon2LS provides an extensive set of visualization tools, such as high performance chart controls.

**Results:**

With a variety of options that include peak processing, deisotoping, isotope composition, etc, Decon2LS supports processing of multiple raw data formats. Deisotoping can be performed on an individual scan, an individual dataset, or on multiple datasets using batch processing. Other processing options include creating a two dimensional view of mass and liquid chromatography (LC) elution time features, generating spectrum files for tandem MS data, creating total intensity chromatograms, and visualizing theoretical peptide profiles. Application of Decon2LS to deisotope different datasets obtained across different instruments yielded a high number of features that can be used to identify and quantify peptides in the biological sample.

**Conclusion:**

Decon2LS is an efficient software package for discovering and visualizing features in proteomics studies that require automated interpretation of mass spectra. Besides being easy to use, fast, and reliable, Decon2LS is also open-source, which allows developers in the proteomics and bioinformatics communities to reuse and refine the algorithms to meet individual needs.

Decon2LS source code, installer, and tutorials may be downloaded free of charge at .

## Background

High resolution mass spectrometry (MS) is used extensively in proteomics and metabolomics studies to identify and quantify proteins and metabolites [1]. This information is inferred from peak patterns observed in either mass spectra of intact proteins, digested proteins (i.e., peptides) or metabolites, or tandem mass spectra (MS/MS) of proteins, peptides, or metabolites fragmented as a result of collision-induced dissociation within the instrument. While hundreds of individual species can be resolved from a single mass spectrum, even relatively simple proteomics and metabolomics samples can result in thousands of overlapping isotopic patterns. As these patterns may not be readily separable by the instrument or discernible by downstream processing algorithms, liquid chromatography and gas chromatography (GC) are often coupled to MS to reduce the complexity of an individual mass spectrum. Biological material eluting from the chromatographic system is continuously transferred to the mass spectrometer during the course of the analysis, and a mass spectrum is captured at regular intervals. As a result, a single experiment may contain thousands of mass spectra that require automated interpretation.

A single mass spectrum is composed of a list of ion mass-to-charge (*m/z*) ratios and their abundance values. As most elements (e.g., carbon, hydrogen, etc.) are naturally present in different isotopic forms, a *population *of the same molecular species produces a pattern that reflects the incorporation of the different isotopic contributions [2]. As a result, a charged species is observed not as a single peak but as a pattern of peaks whose relative heights and *m/z *depend on the isotopic distribution of the elements they are composed of and operational aspects of the instrument such as resolution, type of detector, etc. Thus simply selecting each observed peak as a unique chemical species would give rise to too many false positives. Proper inference of chemical species from the mass spectra requires that the pattern of related peaks be grouped together into unique explanatory isotopic patterns, a process typically referred to as deisotoping. Since carbon, hydrogen, oxygen, nitrogen, phosphorus, and sulphur are the main elemental constituents of most biomolecules, their isotopic distribution in nature (or in the biological material the sample was grown) determines the spacing between observed peaks, as well as the relative heights of the peaks. (In should be noted here that the difference between masses of isotopes of the same element is not the same as the difference in the mass for the different number of neutrons between them because of the mass deficit resulting from nuclear binding energy of each nucleus. Hence, the mass difference between ^13^C and ^12^C, for example, is different from that between ^1^H and ^2^H and from half of the difference between ^34^S and ^32^S. In fact, in high resolution FTICR-MS runs one can discern the contribution of the ^34^S atom to the third isotope, separate from that of other atoms). The isotopic distributions of unmodified peptide species are primarily influenced by carbon as it has the largest proportion of naturally abundant isotope to any alternative isotope (i.e., 98.89% ^12^C, 1.11% ^13^C). Thus, "isotopic" species of peptides have a mass difference on average, of ~1.003 Da (the difference between the masses of ^13^C and ^12^C). The spacing between the *m/z *peaks is approximately equal to 1.003/*charge *and relative peak heights depend on the elemental composition of the peptides. Since the hundreds of differently charged peptides transferred to the mass spectrometer may have abundances that span several orders of magnitude, the resulting mass spectrum can be composed of complex overlapping contributions. As a result, "deisotoping" algorithms are required to collapse a complex mass spectrum into a representative set of peptide (or metabolite) masses (typically the monoisotopic species) and their respective abundance values. It may be noted here that the nature of the spectrum observed is affected by the complexity of the sample and the distribution of masses and charges of the chemical species in the sample. Hence the complexity of deisotoping step depends on these factors. For example, ESI spectra are typically harder to deisotope than MALDI spectra because most peptides carry multiple charges thus requiring the ability to accurately determine charge state.

Several deisotoping and visualization algorithms have been described in the literature [3-9] and are available as part of commercial vendor packages such as XCalibur (Thermo Fisher), MassHunter (Agilent) and Elucidator System (Rosetta Biosoftware). For example, BUDA, which is used to analyze FTICR data, incorporates the algorithm described by Kaur & O'Connor [7] to deisotope intact protein mass spectra. Pep3D[10] is useful for visualizing an LC-MS dataset as a density plot of abundance (as a function of *m/z *and scan), but does not perform deisotoping. MapQuant [11] is used in the PePPer platform [12] to deisotope LC-MS data and has image processing algorithms that utilize the retention time dimension. While XCMS [13] is useful for data pre-processing, peak-detection, retention-time alignment and peak matching across samples, this algorithm also does not perform deisotoping. msInspect [14] is an analysis package for deisotoping high-resolution LC-MS data in mzXML format in sequential steps. Peaks are first detected in each scan (along the *m/z *dimension) using a wavelet additive decomposition method and then peaks identified as eluting isotopes in the retention dimension are retained. Peak clusters are identified as peptides by comparing the heights of observed isotopes to the heights of theoretical isotopes, which are approximated using a Poisson distribution. OpenMS is a recently released pipeline which allows rapid application development for liquid-chromatography mass spectrometry analyses. In addition to providing rich visualization capabilities it also provides implementation of feature discovery algorithms, retention time alignment algorithms and other processing routines. Vendor software systems also provide access to some analytical capabilities. However, the algorithms employed are typically proprietary and specific to vendor systems, preventing standardization across different instrument types. In addition the range of options provided by the vendor software systems is typically limiting for users with fairly specific needs and customization can be challenging if at all possible. For example, while the Thermo Fisher XCalibur system can be used to extract a list of deisotoped species, it can only do so for a limited set of the most abundant features. As a result, users with specific needs such as access to lists of observed peaks, isotopic patterns, chemically labeled pairs etc, typically have to write their own analytical software.

THRASH (Thorough High Resolution Analysis of Spectra by Horn)[15] is one of the most well known and comprehensive algorithm for analyzing mass spectra. It includes methods for calculating background noise levels, determining charge state using the Fourier-Transform/Patterson technique [16], calculating theoretical profiles [2,17], and for subsequent fitting with observed isotopic profiles. While a functional application of THRASH was not provided by its developers, the algorithm was reportedly incorporated into the MIDAS [18] data system. Another application of THRASH was released in the form of ICR2LS (available only in executable format; G.A. Anderson, ). We have recently modularized the deisotoping and other algorithms previously implemented in ICR2LS (developed in Visual Basic 6 and unpublished) into an open-source software package referred to as Decon2LS, which has been developed in C++ with several improvements to optimize performance by almost an order of magnitude.

Herein, we describe Decon2LS, a software package for finding and visualizing features in high resolution MS datasets. Decon2LS uses a derivative of THRASH to determine monoisotopic mass lists from a given dataset of *m/z *and intensity values across scans and supports several different file formats for data visualization, including Thermo Fisher .RAW files, Agilent TOF .wiff, Micromass .dat files and Bruker .ser files provided the libraries are installed. In addition, mzXML standard and ascii file formats are supported, which provides users with a single tool for processing high resolution data from all major MS formats. Decon2LS has already been applied extensively in our laboratory to analyze >15,000 datasets obtained using different types of high resolution mass spectrometers. While the THRASH algorithm was developed for mass spectra of intact proteins and Decon2LS may be used for this purpose with a suitable adjustment of parameters, the implementation is geared towards deisotoping of spectra of proteins and peptides of mass less than 10,000 Da.

## Implementation

### Basic Architecture

**Figure 1:**
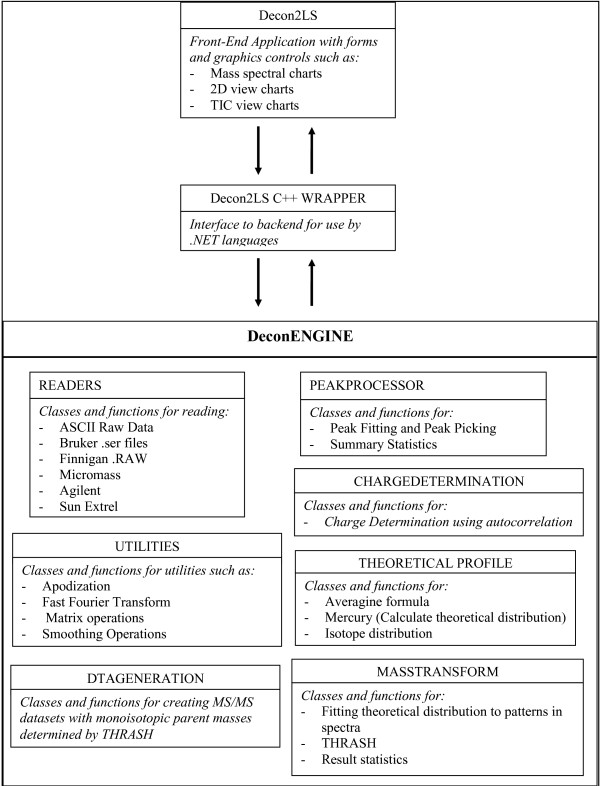
**Architecture of the Decon2LS software: Decon2LS is written using a mixture of visualization modules written in C# in the .NET programming environment, and backend libraries (DeconEngine) written in standard C++ libraries**. The DeconEngine library implements modules for reading different types of vendor formats, peak finding routines, theoretical isotopic pattern generation and deisotoping. The front end talks to these libraries through wrappers written in Microsoft's Managed C++.

The basic architecture of the Decon2LS software package is depicted in Figure 1. Written on the .NET framework, the core of Decon2LS (called the DeconEngine) is written in standard C++ using Standard Template Libraries (STL). The user interface is written in C# with a C++ wrapper that acts as the interface between front-end and back-end processing. For Windows machines, Decon2LS requires installation of the .NET framework (default for XP and higher). A separate installer that installs both Decon2LS and the .NET framework is also provided to accommodate older operating systems. Installation details and the source code are available for download free of charge at . Access to the source code allows Unix-based computer users to detach the engine and build their own wrappers and user interface. The Apache open-source license allows the original code to be modified for individual needs.

### Algorithm

A typical mass spectrum of a proteomics sample consists of patterns of isotopic peak distributions for many different peptides, each with its own charge and intensity that can vary over five orders of magnitude. The pattern for a singly charged peptide is made up of several peaks spaced ~1 Thompson (unit of *m/z*) apart, representing a mass difference of ~1 amu. (In reality, the individual "isotopic" peaks are composed of "fine structure" peaks, representing the slight mass differences for the different possible isotopic combinations of the elements, which are not generally resolvable by the mass spectrometer). The same peptide, but with a higher charge generates peaks with similar relative intensities as in the single charge state, but with spacing that is ~1.003/charge. Determining the masses present in a spectrum is a challenging task that requires selecting peak patterns most likely to represent peptides and metabolites in the spectrum. The Decon2LS software package uses an in house modified version of the THRASH algorithm to select these peak patterns.

**Figure 2:**
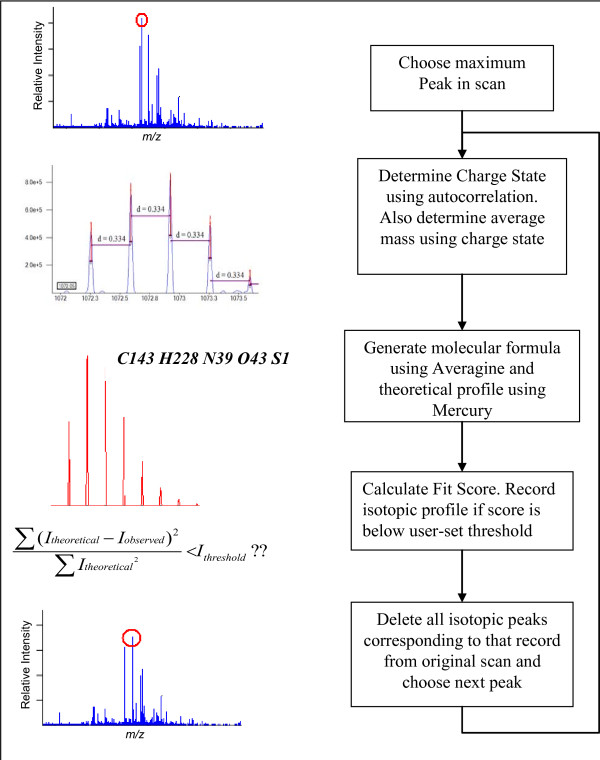
**The variant of the THRASH algorithm used in Decon2LS: peaks in the mass spectrum are found by comparing their intensity to their shoulders and to the background**. Starting at the most intense peak deisotoping is performed by determining charge state, estimating empirical formula, generating a theoretical profile, scoring the theoretical profile and deleting points from the spectrum. This process is continued while peaks of intensity greater than a user specified threshold ratio of the background still exist.

The THRASH algorithm uses modules to determine peak charge states (the autocorrelation algorithm [16]), generate theoretical profiles (the Mercury algorithm [2]) and to score theoretical profiles against observed patterns. Decon2LS implements a derivative of the THRASH algorithm, using the same components, but with different scoring schemes. Indexing and caching algorithms (described below) are also used internally to optimize algorithm performance. The steps performed by the THRASH algorithm variant used in Decon2LS are depicted in Figure 2, and described further in the text that follows. Note that the algorithm can either be run interactively on an individual mass spectrum or in a batch mode on all the mass spectra in a dataset using the Process form available in the application.

1. Peaks are picked from the mass spectrum provided the signal-to-noise ratio (calculated as the ratio of the intensity of the peak to the average intensity at the valleys of the peak) is greater than a user-specified threshold. In addition, the intensity of the peak is required to be greater than a user-specified multiple of the background intensity computed for the spectrum. This background intensity is computed for the entire spectrum as the average intensity of those points that are within five standard deviations of the average of *all *points in the spectrum. The selected peaks are processed in steps 2–6.

2. While unprocessed peaks remain in the spectrum, the charge of the most intense unprocessed peak is determined using the autocorrelation algorithm [16], and the average mass is computed from the *m/z *of the peak and the computed charge.

3. The most likely empirical composition is determined using the average mass determined in step 2 and the Averagine algorithm [17], which assumes an empirical composition that is equal to the average composition of all the peptides in a protein FASTA file. By default, Decon2LS uses the average formula derived from the Swiss-Prot protein FASTA database.

4. A theoretical spectrum is generated using the Mercury algorithm [2] for the empirical formula generated in step 3 and fit against the observed spectrum by aligning the most abundant peak of the theoretical spectrum to the peak under consideration after scaling each peak to an intensity of 100. The score for the fit is computed on the basis of the similarity of the theoretical pattern to the observed pattern, using one of the following three user selectable functions: a. Area fit function:

$$\frac{\sum_{j:I_{j}^{theo}>c}(100\frac{I_{j}^{obs}}{I_{\max}^{obs}}-I_{j}^{theo}{)}^{2}}{\sum_{j:I_{j}^{theo}>c}{\left(I_{j}^{theo}\right)}^{2}}$$

b. Peak fit function:

$$\frac{\sum_{j:j^{th}\text{point is isotope peak: }I_{j}^{theo}>c}(100\frac{I_{j}^{obs}}{I_{\max}^{obs}}-I_{j}^{theo}{)}^{2}}{\sum_{j:I_{j}^{theo}>c}{\left(I_{j}^{theo}\right)}^{2}}$$

c. Chi-square fit function, as specified in Senko, et al. [17].

$I_{j}^{theo}$ represents the intensity of the *j*^*th *^point in the theoretical mass spectrum of the empirical formula estimated from the peak mass and the averagine formula, $I_{j}^{obs}$ is intensity of the corresponding *m/z *value in the observed mass spectrum, and $I_{\max}^{obs}$ is the intensity of the observed peak that the most abundant peak of the theoretical isotopic profile is matched to. Because spacing between points in a theoretical profile generated from Mercury is uniform, while the spacing between points in the observed spectrum is not, $I_{j}^{obs}$ values must be estimated by fitting a natural cubic spline to the observed spectrum and then using this spline to interpolate intensities at evenly spaced *m/z *values of the theoretical profile. *c *is a user-specified intensity threshold that a point needs to meet in order to be considered in the scoring function. This threshold prevents low level noise from being factored into the fitness score. Instrumental factors can influence the isotopic distributions and these factors differ for various instrument types. These factors influence the fitness score and must be considered when setting fitness thresholds.

5. Alternative fits are also computed by aligning the most abundant peak of the theoretical pattern with the observed isotopic peaks ("THRASHing"), which is accomplishing by moving the theoretical pattern 1.003 Da (using charge to convert Da to Thompson units). The user is able to choose between THRASHing to the next isotopic peak only as long as scores improve and as long as a peak is found at the appropriate Thompson distance away (Complete Fit). The highest of the fit values is maintained for further consideration.

6. If an acceptable fit is found, then the isotopic peaks of the observed peak are deleted and the points in the spectrum are set to 0. The number of isotopic peaks deleted is specified indirectly by a user-specified Deletion parameter that specifies the minimum relative abundance (compared to most abundant isotope) of all isotopic peaks to be deleted. Monoisotopic mass of the feature is calculated as the monoisotopic mass of the theoretical spectrum when overlaid with the best-fit isotope peak.

7. If an acceptable fit is not found, then the current peak is removed from the list of unprocessed peaks and the process is repeated, starting with step 2.

Because the nature of the theoretical isotopic profile does not change much over the range of 1 Da mass, we optimized the performance of the algorithm by caching the isotopic distribution at every integer mass by only storing the position and the relative intensity of isotopic peaks. The theoretical profile for a given mass and resolution can then be created from the cached intensities and masses of isotopic peaks by super imposing appropriate peak shapes on them.

### Visualization

**Figure 3:**
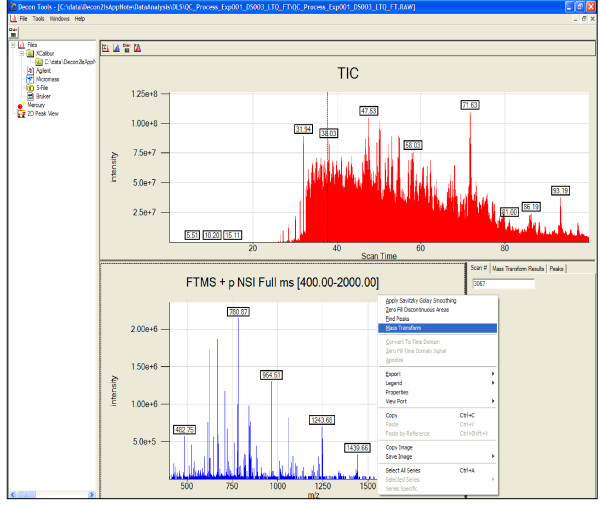
**A screen-shot of the traditional TIC-based view in Decon2LS of dataset ShewFed028_LTQFT_1_29May05_Andro_0505-02: The top pane shows the total-ion-chromatogram for the dataset, while the bottom pane shows the mass spectrum of the scan number 2067 that is selected in the TIC pane**. The bottom right pane shows details of peak finding and deisotoping operations.

**Figure 4:**
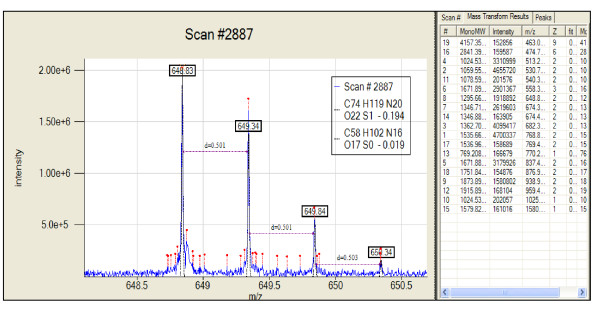
**Detailed description of peak-finding and deisotoping results on spectrum number 2887 from dataset Caulo_084_run3_03Nov06_Andro_06-06-12 can be seen in the spectral pane**. In this figure we can see the theoretical isotope distribution overlapped on top of the observed pattern.

Two main forms of visualization are provided to navigate datasets and the results generated. The first is a "traditional" view (Figure 3) where the total ion chromatogram for the analysis is displayed in a navigation pane on the top and is linked with the mass spectrum for a chosen scan on the bottom pane. By right-clicking on the bottom pane, one can choose the desired operations. For example, the results from deisotoping a single scan (Figure 4) can be viewed by clicking on any of the transformed records. The results include details such as the overlap of theoretical and observed spectrum, the charge state of the ion, the molecular formula generated by Averagine, and the monoisotopic peak. Options also are available to view and process contiguous spectra summed across a specified window.

**Figure 5:**
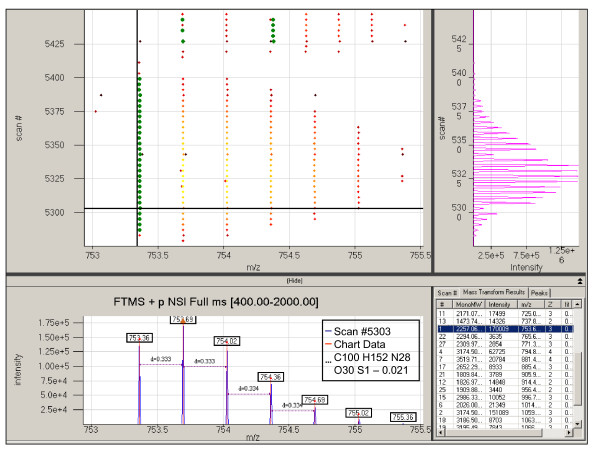
**The heat-map view of the data seen for dataset EIF_NAF_CF_Cys1_21Oct06_OWL_06-08-02 between m/z range of 550 and 1050 and scan range of 5275 and 5450**. The top pane shows the heatmap of the data while and illustrates the complexity of some regions of an LC-MS experiment. The bottom pane shows the spectrum selected in the heatmap and the right pane shows the selected ion chromatogram of the chosen m/z with a tolerance of 0.1 Thomson.

The second form allows users to visualize results generated from a deisotoping or peak finding analysis. In this view (Figure 5), navigation is provided with a central two dimensional heat map of all peaks found in an LC-MS dataset. The central pane is linked to the mass spectrum for a chosen scan on the bottom pane, and the selected ion chromatogram for a chosen *m/z *on the right pane. The mass spectral pane allows users to perform processing on the linked mass spectrum through a context menu.

### Parameters

Parameters required to control all functionalities can be visualized through an options form in Decon2LS. The main parameters include: options for specifying the processing parameters used in an analysis e.g. type of peak-fitting model (apex, three point quadratic, lorentzian); signal-to-noise thresholds; an averaging window size to sum spectra along the retention time dimension to enhance low intensity isotopic patterns seen over multiple scans; fitness score thresholds to control the rate of false positive features;, scan and *m/z *range applicable to running the algorithms; spectral pre-processing options for zero-filling and smoothing using the Savitzky- Golay filter; and the ability to change the isotopic composition of naturally occurring isotopes. All parameters can be saved in an XML format for future use.

### Output

For each dataset that is processed in batch mode, Decon2LS generates the following three output files:

1. **Features file **([dataset]_isos.csv). This comma delimited file provides details for all deisotoped features, such as monoisotopic mass, most abundant isotope intensity, scan number, fitness score, and other feature-relevant information.

2. **Scan summary file **([dataset]_scans.csv). This comma delimited file contains summary statistics for the LC scans in the datasets such as the scan type (MS or MS/MS), the base peak *m/z*, the total-intensity-chromatogram value per scan, number of peaks present, and number of peaks deisotoped.

3. **Peaks file **([dataset].dat). This file contains relevant binary data such as the peak information and the deisotoped records that are visualized using the two dimensional view.

The output of Decon2LS can be loaded into auxiliary software such as VIPER [19], which identifies peptides by comparing LC-MS mass and elution time features to peptides in a database constructed from previous LC-MS/MS analyses [20].

### Other features

**Figure 6:**
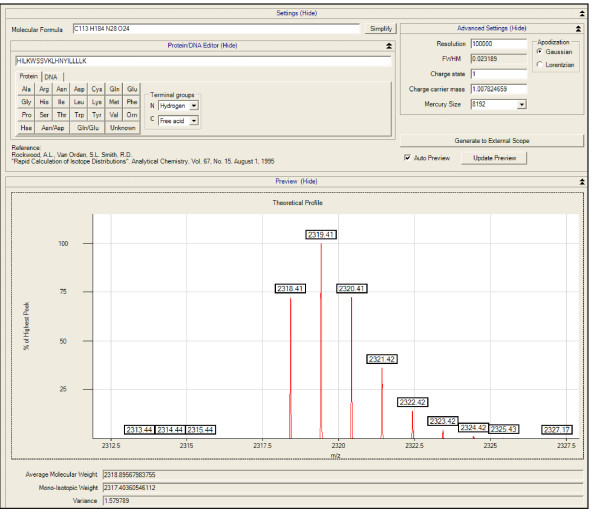
**The Mercury form provides an implementation of the Mercury algorithm to allow users to generate theoretical isotopic distribution for any compound on the basis of the elemental isotopic distribution using user specified parameters for charge state, resolution and type of peak shape**. In this figure we show the theoretical isotope pattern of C113 H184 N28 O24 with charge of 1 generated at a resolution of 100,000.

Decon2LS includes a number of other data processing features. For example, the Mercury algorithm can be used to view the theoretical spectrum of any peptide sequence or atomic formulae at different charge states and instrument resolution (Figure 6). Decon2LS also creates TIC plots from the Scan summary file. Additionally, the software can be used to process tandem MS results by using the DeconMSn algorithm to create ".dta" spectrum files for a given LC-MS/MS dataset [21]. These files can be processed using search-engines such as SEQUEST[22] to identify peptides.

### Reusable Modules

With a modular design and open source license, Decon2LS allows users to extrapolate necessary modules based purely on functionality for individual use. A good example of this reusability aspect is the DeconMSn tool [21] that uses the raw data readers, and the peak-finding and THRASH routines (including Averagine and Mercury) that are incorporated as part of the DeconEngine library to process LC-MS/MS datasets. X2XML (available at ) is another tool that uses the multiple raw data readers to convert several file-formats to the mzXML standard for representing data. The visualization routines and user controls may readily be plugged into other .NET applications.

## Results and discussion

To date, Decon2LS has been applied to process > 15,000 datasets in our laboratory. Three of these datasets from unrelated biological samples analyzed on different high resolution instruments are used for the purpose of the following discussion. Each of these datasets was processed in batch mode, using Decon2LS to deisotope the mass spectra. Additional details regarding these datasets are provided in Table 1[23]. All three datasets are available for download at .

**Table 1:** Description of the samples analyzed by Decon2LS for this study

**Sample Name**	**Species**	**Instrument Description**	**Sample Description**
**ShewFed028_LTQFT_1_29May05_Andro_0505-02**	*Shewenella oneidensis *MR-1	Thermo Fisher LTQ-FT linear ion trap-FTICR hybrid mass spectrometer with electrospray ionization (ESI)	A chemostatic growth experiment in oxygen-limited condition with the addition of fumarate (an electron-acceptor)

**Caulo_084_run3_03Nov06_Andro_06-06-12**	*Caulobacter crescentus*	11.5-Tesla FTICR instrument, designed and constructed in our laboratory at PNNL [23]	Insoluble preparation of cells that were carbon-limited (glucose limitation) as part of a time course study

**EIF_NAF_CF_Cys1_21Oct06_OWL_06-08-02**	*Homo sapiens*	Thermo Fisher LTQ-Orbitrap hybrid mass spectrometer with electrospray ionization (ESI)	Samples from human nipple aspirate fluid collected as part of bilateral studies of possible ductal carcinoma in breast tissue

Three samples generated from three different species and run on three different high resolution instruments were used to assess the results produced by Decon2LS. These datasets will be available for download at .

**Figure 7:**
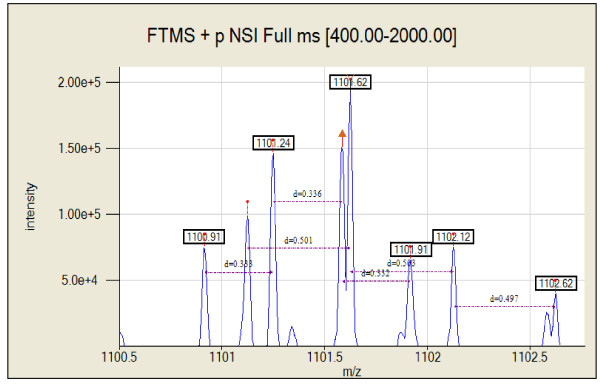
**The figure shows a zoomed in view of scan # 9520 in dataset ShewFed028_LTQFT_1_29May05_Andro_0505-02 where two different chemical species produce isotopic peaks that overlap**. Decon2LS draws line between isotopic peaks of the same species and shows how the two species were successfully deisotoped.

A common occurrence while deisotoping a mass spectrum is that two or more peptides have overlapping isotopic patterns, which can result in poor fitness scores. The ability of Decon2LS to accurately determine the monoisotopic mass of two overlapping species whose peaks overlap with one another is exemplified in Figure 7. In this example two isotopic distributions are detected; a 2201.233 Da species at charge state 2 and a 3300.725 Da species at charge state 3. These two species resulted in two isotopic peaks separated by only 0.044 *m/z *units, as can be seen in Figure 7 the result is two partially overlapping peaks. While not perfect, the ability to resolve overlapping patterns is enhanced by the sequence in which deisotoping attempts are performed by the algorithm. For example, peaks are processed in order of descending intensity, so the highest peak of an overlapping isotopic distribution is processed first. However, during the first attempt, the fitness score may be too low. In this case, the algorithm proceeds to deisotope other patterns in the spectrum. Once Decon2LS succeeds in processing one of the overlapping patterns, the software removes the processed peak from the spectrum. At this point, the process of deisotoping the original pattern starts over at one of the remaining isotopic peaks, and after "THRASHing" the best match between the theoretical pattern and the remaining observed pattern is determined. Developing methods to tag overlapping isotopic patterns and fit them using least-squares based procedures, such as those described elsewhere [24] may further improve algorithm performance.

The processing parameters used for the three datasets are listed in Table 2 and details of the corresponding "isos" and "scans" files created for each dataset are given in Table 3. 171,725 patterns were determined from 2,967,743 peaks in the *Shewanella oneidensis *MR-1 sample, while 94,813 patterns (from 5,797,366 peaks) and 44,000 patterns (from 1,556,780 peaks) were observed for the *Caulobacter crescentus *and *Homo sapiens *samples, respectively. Note that the processing time for Thermo Fisher LTQ-FT and LTQ-Orbitrap hybrid datasets is typically less than the processing time for datasets obtained on the 11.4T FTICR (constructed in house) as the hybrid datasets were acquired in thresholded mode, which makes it harder to deisotope low intensity patterns because background intensity levels are harder to determine and low intensity isotope peaks may effectively "disappear" upon thresholding. Nevertheless, Decon2LS was able to deisotope features that were discernible to trained mass spectrometrists. Although the datasets can be acquired in non-thresholded mode, files larger than 2 GB cannot currently be read by the Xcalibur libraries.

**Table 2:** Main processing parameters and values used in analysis

Category	Parameter	Function	ShewFed028_LTQFT_1_29May05_Andro_0505-02	Caulo_084_run3_03Nov06_Andro_06-06-12	EIF_NAF_CF_Cys1_21Oct06_OWL_06-08-02
Peak Picking	Peak-Fit Type	Sets the type of peak-fitting to be performed (APEX, LORENTZIAN or Quadratic)	Quadratic	Quadratic	Quadratic
	
	S/N	Sets the signal-noise ratio as the ratio of maximum peak intensity to the minimum of floor intensities on either side of the peak	3	3	3
	
	Minimum Background Ratio	Sets the maximum intensity level to be considered as background	3	3	3

Horn Transform Parameters	Charge mass	Mass of charge carrier	1.007276	1.007276	1.007276
	
	Max mass	Maximum mass to consider	10000	10000	10000
	
	Max charge	Maximum charge to consider	10	10	10
	
	Set_THRASH	If set, scores each isotopic profile in stops of +/- 1 Da for fit to data, exits and returns if new_score > current_score	True	True	True
	
	Set_Complete_Fit	If set, works same as THRASH except the best fit from a series of fits is returned	False	False	False
	
	Distribution-Fit Type	Sets the method of fitting theoretical and observed distributions (Area, Peak or Chi-Squared)	Area	Area	Area
	
	Allowable shoulders	Sets the number of allowable shoulders as the number of non-decreasing peaks preceding a minima for it to be considered a shoulder	1	1	1
	
	Max_Fit	Range 0 to 1, measure the maximum difference allowable between theoretical and observed distribution	0.15	0.25	0.15
	
	Threshold intensity for score	Sets the minimum normalized intensity (0-100) for selection of the area of the peak that will be used for fit calculation	10	10	10
	
	Threshold intensity for deletion	Sets the intensity threshold (normalized 0 – 100) that determines which areas of a peak are to be deleted.	1	1	1

The typical parameters and values used by Decon2LS for processing data files from the three different instruments are shown. Peaks were required to display a S/N of at least 3, while also being at least 3 times as intense as the background noise. Horn transform is typically performed with the AreaFit function using a threshold fitness score of 0.1 (0 being a perfect match).

**Table 3:** Results of processing three LC-MS datasets using Decon2LS

Dataset	MS scans in dataset	peaks in dataset	isotopic patterns found	LC-MS features	Processing time
ShewFed028_LTQFT_1_29May05_Andro_0505-02	5100	2,967,743	106,150	6670	22 min

Caulo_084_run3_03Nov06_Andro_06-06-12	2950	5,797,366	76,567	5036	1 hr 08 min

EIF_NAF_CF_Cys1_21Oct06_OWL_06-08-02	3312	1,556,780	34,205	2817	18 min

This table lists the numbers of scans in the LC-MS datasets, the numbers of peaks found by the peak detection algorithm, the number of isotopic patterns found in the peaks and the number of LC features found by grouping together the isotopic patterns using retention time. The processing time was larger for the in-house FTICR dataset because the dataset was larger (~8 Gb) than the other two datasets, which were smaller because the Thermo Fisher acquisition software, Xcalibur, sets a minimum intensity threshold.

Once all ions were deisotoped, the software tool VIPER [19] was used to find LC-MS features by grouping deisotoped features of similar masses in neighbouring LC scans into a single mass and retention time feature. These LC-MS features can be used to identify proteins and metabolites [20].in proteomics and metabolomics studies [25].

## Conclusion

Decon2LS, a new software package for automated processing and visualization of LC-MS datasets supports multiple vendor formats, making it useful for analyzing data from different MS instruments. Datasets can be viewed in traditional mode, whereby a TIC can be used to select which scan of the analysis to view in a mass spectrum pane. A two dimensional view is also supported in which a contour map of peaks is linked to a selected ion chromatogram and a mass spectrum pane that aids users in choosing correct deisotoping parameter values.

Decon2LS uses a variant of the THRASH algorithm to determine accurate monoisotopic masses for the vast majority of observed isotopic distributions. The deisotoped results can be viewed by overlaying theoretical patterns on the observed spectrum so that the user has feedback on how the deisotoping worked for a particular dataset. Through the use of indexing data structures and faster search routines, Decon2LS is an order of magnitude faster than ICR2LS.

## Availability and requirements

**Project Name: **Decon2LS

**Project Home Page: **

**Operating System: **Microsoft Windows XP.

**Programming Language: **Algorithms in C++, Visualization in C#

**Other Requirements:**.NET framework 1.1 for operation, Visual Studio 2003 for compilation

**License: **Apache 2.0

**Any Restrictions to use by non-academics: **None

## Authors' contributions

NJ dissected ICR2LS code, architected Decon2LS visualization components and back-end, developed algorithms for improved performance and wrote the back-end processing components. AM added several features to Decon2LS related to both visualization of parameters and analytical components of the back-end. He also played a key role in several crucial bug fixes of the core algorithm. KL wrote the visualization controls that were used in Decon2LS and some of the forms used in the program. JNA provided scientific guidance. GAA was the architect and developer of ICR2LS, the software that Decon2LS draws the underlying algorithms (excluding the caching and visualization approaches) from. RDS provided crucial scientific guidance and leadership to the development group.
